# Surgical Pulmonary Embolectomy Versus Systemic Thrombolysis in High-Risk Pulmonary Embolism: A Retrospective Single-Center Analysis

**DOI:** 10.3390/jcm14207448

**Published:** 2025-10-21

**Authors:** Arash Motekallemi, Linus C. Markus Rohrwild, Jonas Ajouri, Ridvan Dryana, Tamari Tvildiani, Verena Vach, Ralf M. Muellenbach, Ali Asghar Peivandi

**Affiliations:** 1 Department of Cardiac Surgery, Campus Kassel of the University of Southampton, Mönchebergstraße 41-43, 34125 Kassel, Germany; ridvan.dryana@gmail.com (R.D.); tamari.tvildiani@gnh.net (T.T.); aliashgar.peivandi@gnh.net (A.A.P.); 2 Department of Cardiothoracic Surgery, University Hospital Muenster, 48149 Muenster, Germany; 3 Kassel School of Medicine, Campus Kassel of the University of Southampton, 34125 Kassel, Germany; lr4g22@soton.ac.uk; 4 Department of Anesthesiology and Critical Care Medicine, Campus Kassel of the Philipps University, Marburg, 35032 Kassel, Germany; jonas.ajouri@gnh.net (J.A.); verena.vach@gnh.net (V.V.); ralf.muellenbach@gnh.net (R.M.M.); 5 Department of Anesthesiology and Intensive Care Medicine, University Hospital Giessen and Marburg, and Philipps University Marburg, 35032 Marburg, Germany

**Keywords:** pulmonary embolism, high-risk patients, surgical pulmonary embolectomy, systemic thrombolysis, clinical outcomes

## Abstract

**Background:** Pulmonary embolism (PE) is a life-threatening condition with high mortality, particularly in high-risk cases where rapid clinical deterioration is common. The optimal management strategy for high-risk PE remains debated. Systemic thrombolysis (ST) is widely used but is associated with substantial bleeding risks. Surgical pulmonary embolectomy (SPE) has re-emerged as a viable alternative, particularly in patients with contraindications to thrombolysis or failed response. However, the evidence comparing SPE and ST in critically ill patients remains limited, and current guidelines provide only limited guidance. This study aims to evaluate the outcomes between SPE and ST in critically ill patients, focusing on mortality and complication rates. **Methods:** This retrospective study included 96 high risk patients with severe acute pulmonary embolism treated between 2015 and 2023, with 48 undergoing SPE and 48 receiving ST who were matched 1:1 based on baseline variables and hemodynamic presentation. Outcomes assessed included in-hospital mortality, PE-related death, neurological complications, bleeding events, hospitalization duration, as well as further postinterventional complications. **Results:** In-hospital mortality was 16.6% in the SPE group in contrast to 25.0% in the ST group (*p* = 0.765). Neurological complications were significantly lower in SPE (2.1%) compared to ST (12.5%) (*p* = 0.05). Life-threatening hemorrhage occurred at similar rates in both groups (SPE: 18.8%, ST: 14.6%); however, non-life-threatening bleeding was more common in ST (16.7% vs. 2.1%, *p* = 0.014). Hospitalization duration was significantly longer for SPE patients (mean 17.4 vs. 11.4 days, *p* < 0.001), who also presented with more severe disease, including higher ECMO utilization. **Conclusions:** SPE is a safe and effective alternative to ST in PE, offering comparable mortality, fewer neurologic complication and a reduced risk of bleeding. These findings highlight the importance of individualized, risk-adapted treatment pathways and support the inclusion of SPE as a frontline consideration in the management of PE in critically ill patients in experienced centers with multidisciplinary support.

## 1. Introduction

Pulmonary embolism (PE) is a potentially life-threatening condition with approximately 90,000 deaths annually across Europe and the United States [1]. High-risk cases present a critical challenge due to their rapid clinical deterioration. The prognosis of untreated PE is poor, with a case-fatality rate of 30%. Fortunately, with early recognition and the implementation of appropriate therapeutic strategies, mortality can be reduced [2].

Despite advancements in several medical fields (i.e., imaging modalities, risk stratification tools and the widespread use of effective anticoagulants), the optimal management strategy for high-risk PE remains controversial [3,4]. The cornerstone of treatment in these cases is timely reperfusion therapy aiming to restore pulmonary perfusion, reducing RV afterload, and preventing hemodynamic decompensation. Systemic thrombolysis (ST) involving intravenous administration of fibrinolytic drugs has long been considered the first-line therapy for patients with massive PE who have no contraindications to thrombolytic agents [4,5]. While ST is effective in rapidly dissolving clots and improving hemodynamics, it carries significant risk, particularly of major bleeding. Hemorrhagic stroke is the most common complication, and rates of clinically significant bleeding events have been reported as high as 20% in some series [5,6,7]. This risk is further amplified in elderly patients or those with prior surgery or cerebrovascular disease.

Surgical pulmonary embolectomy (SPE) has re-emerged as a valuable alternative, especially in patients with contraindications or failing response to ST. Historically viewed as a measure of last resort, SPE has undergone a renaissance due to technical improvements in cardiopulmonary bypass, perioperative care, and increasing experience at high-volume centers.

Recent literature shows that SPE can not only be applied safely but can serve as a feasible real-world alternative in complex cases compared to ST [8,9,10,11]. While SPE is often used in more critically ill patients, it is also associated with higher in-hospital mortality compared to ST. However, this may be due to patient selection and severity rather than the intervention itself [12,13,14,15]. Furthermore, there is no clear consensus regarding which patient populations derive the greatest benefit from surgery versus systemic treatment. Current European Society of Cardiology (ESC) and American Heart Association (AHA) guidelines acknowledge the role of SPE but stop short of offering detailed algorithms for its use beyond thrombolysis failure or contraindications [16,17,18].

Given the current gaps in the literature, this study aims to analyze our institutional experience with SPE and ST. The primary goal was to determine whether SPE offers a more favorable risk-benefit profile than ST, especially in critically ill patients with significant hemodynamic instability.

## 2. Materials and Methods

### 2.1. Study Design and Setting

This retrospective cohort study was conducted at Klinikum Kassel, Germany, and included patient data collected between 1 January 2015, and 31 December 2023. Patients diagnosed with acute pulmonary embolism (PE) were identified through the institutional electronic medical records system (MEDICO) using ICD-10 codes. Given the known limitations of ICD-10 coding for PE identification—such as potential under-detection and coding errors—case ascertainment was performed in accordance with recent validation studies and, where possible, supplemented by review of imaging reports to enhance accuracy [19].

### 2.2. Eligibility Criteria

Patients were eligible for inclusion if they were between 18 and 101 years of age and had a radiologically confirmed diagnosis of high-risk acute PE, as defined by the presence of hypotension (systolic blood pressure < 90 mmHg), shock, or vasopressor dependency, confirmed by computed tomography pulmonary angiography (CTPA).

Exclusion criteria comprised:

- Anticipated life expectancy < 6 months due to advanced comorbidities,
- Pregnancy,
- Pre-existing chronic thromboembolic pulmonary hypertension,
- Recent major surgery (within 7 days of PE diagnosis),
- Diagnosed coagulopathies contraindicating either thrombolysis or surgical intervention.

### 2.3. Data Collection and Variables

Clinical and procedural data were manually extracted by two independent reviewers using a predefined case report form. Data included baseline demographics, cardiovascular risk factors, comorbidities, presenting hemodynamic status, laboratory parameters, CTPA findings, treatment modality (systemic thrombolysis or surgical pulmonary embolectomy), use of extracorporeal membrane oxygenation (ECMO), and clinical outcomes.

The primary outcome was in-hospital all-cause mortality. Secondary outcomes included:

- PE-specific mortality (defined as death directly attributable to embolic burden or associated hemodynamic compromise),
- Major adverse cardiovascular events (MACE),
- Neurological complications (defined as new-onset persistent neurological deficit or intracranial hemorrhage),
- Life-threatening and non-life-threatening bleeding episodes,
- Post-interventional complications including AKI, wound infection, hemodynamic instability (including HF and cardiogenic shock), and allergic reactions
- Hospital length of stay

### 2.4. Statistical Analysis

Continuous variables were summarized as means with standard deviations (SD) and compared between groups using unpaired two-sided Student’s *t*-tests. Categorical variables were described as frequencies and percentages and analyzed using Chi-square or Fisher’s exact tests, as appropriate. A two-tailed *p*-value of ≤0.05 was considered statistically significant. All analyses were conducted using IBM SPSS Statistics version 28.0 (IBM Corp., Armonk, NY, USA).

To mitigate confounding and selection bias, patients receiving ST and those undergoing SPE were matched 1:1 based on baseline variables, including age, sex, comorbidity burden, and hemodynamic presentation. Propensity score matching was not employed; however, strict inclusion criteria and standardized data extraction procedures were implemented to approximate clinical equipoise between cohorts.

## 3. Results

### 3.1. Baseline Characteristics

Patient demographics are presented in Table 1. A total of 96 patients fulfilled the inclusion criteria and were enrolled in this study; of these, 48 patients underwent SPE and 48 ST. The mean age across the entire cohort was 65.8 years (SD ± 17), with no significant difference observed between groups (SPE: 66.1 ± 16; ST: 65.6 ± 18 years). Gender distribution was evenly balanced, with 48 males and 48 females included (SPE: 21M & 27F; ST: 27M & 21F). Comorbidities and risk factors were similarly distributed between cohorts and are summarized in Table 1. The prevalence of chronic cardiopulmonary disease (e.g., chronic obstructive pulmonary disease, atrial fibrillation, prior myocardial infarction) was 17.7% (*n* = 17), cases of prior malignancy were recorded at 18.7% (*n* = 18), and obesity was documented in 16.7% of patients (*n* = 16). The presence of deep vein thrombosis (DVT) was noted in 45.8% (*n* = 44) of patients, with equal distribution between the SPE and ST groups. Other risk factors such as type 2 diabetes mellitus (*n* = 11), and history of smoking (*n* = 8) were also evenly distributed between both cohorts.

CPR was administered to 22.9% (*n* = 11) of SPE patients and 33% (*n* = 16) of ST patients pre-intervention. Pre-ECMO was present in 4% of SPE (*n* = 2) patients and 0% of ST patients. Furthermore, the majority of patients in both cohorts presented with acute pre-interventional Cor Pulmonale with moderate RV Dysfunction, defined as TAPSE 10–15 mm, S-prime (Sm) 6–9 cm/s. The distribution of the localization of PE was also homogenous between both cohorts. Indicating an even distribution of acute massive PE cases between both cohorts.

No significant differences were identified in the distribution of comorbidities, indicating adequate baseline comparability between cohorts.

### 3.2. Clinical Outcomes

Clinical outcomes are presented in Table 2.

#### 3.2.1. Mortality and Neurological Events

In-hospital all-cause mortality was observed in 8 patients (16.6%) of the SPE group and in 12 patients (25.0%; *p* = 0.765) of the ST group. Pulmonary embolism-specific mortality, defined as death attributable to persistent embolic burden or right ventricular failure, occurred in 2 SPE patients (4.2%) and 5 ST patients (10.4%; *p* = 0.140). Other causes of death included multiorgan failure (*n* = 1) and stroke (*n* = 6) for the non-surgical cohort. Severe circulatory failure (*n* = 4) and lung infection (*n* = 1) were other mortality causes for the surgical cohort.

Neurological complications, including stroke or persistent neurologic deficit, were significantly less frequent in the SPE cohort, with only one case (2.1%) compared to six cases (12.5%) in the ST group (*p* = 0.05). All neurological events in the ST group were secondary, highlighting the bleeding-related risk profile associated with systemic thrombolytic therapy.

#### 3.2.2. Hemorrhagic Events

Rates of life-threatening hemorrhage—defined as hemorrhage requiring surgical intervention, transfusion of ≥2 units of packed red blood cells, or contributing to hemodynamic instability—were similar between groups (SPE: 9/48 [18.8%] vs. ST: 7/48 [14.6%]; *p* = 0.779). However, non-life-threatening hemorrhage (e.g., hematoma, oozing at puncture sites, minor mucosal bleeding) occurred significantly more often in patients receiving ST (16.7%) compared to SPE (2.1%; *p* = 0.014).

#### 3.2.3. Additional Post-Procedural Complications

The overall frequency of post-procedural complications was high in both groups: 45.8% (*n* = 22) in SPE and 50.0% (*n* = 24) in ST. Hemodynamic instability, manifesting as persistent cardiogenic shock or persistent right sided heart failure was more common in the SPE group (*n* = 12; 25.0%) compared to the ST group (*n* = 5; 10.4%).

Conversely, the incidence of acute kidney injury (AKI), defined by KDIGO criteria, was higher among patients in the ST cohort (*n* = 8; 16.7%) relative to those undergoing SPE (*n* = 4; 8.3%). A total of 44% of patient obtained a lower respiratory tract infection, with patients in the SPE cohort being 6% more likely to develop an infection compared to the ST cohort (25% vs. 18.75%). Pleuritis and pneumonia were most noted.

#### 3.2.4. Hospital Length of Stay

**Figure 1:**
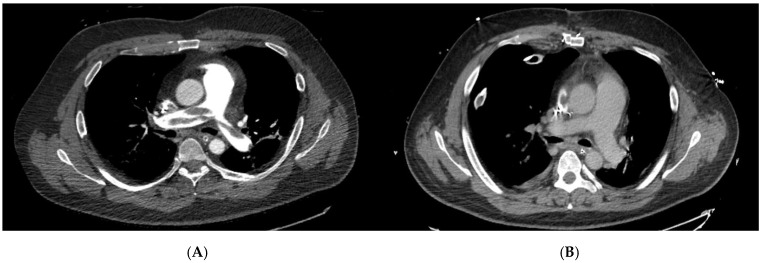
Pre- and postoperative computed tomography pulmonary angiography (CTPA) images of a patient undergoing surgical pulmonary embolectomy (SPE). (**A**) Preoperative axial CTPA demonstrating extensive central pulmonary embolism involving the main pulmonary artery with marked luminal obstruction. (**B**) Postoperative CTPA after surgical embolectomy showing complete removal of thrombotic material and restoration of pulmonary artery patency. These representative images illustrate the morphological success of SPE in a high-risk patient and highlight the immediate anatomical effect of surgical reperfusion.

**Figure 2:**
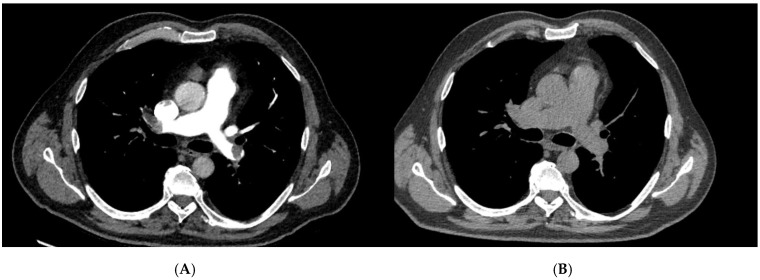
Pre- and post-treatment computed tomography pulmonary angiography (CTPA) of a patient treated with systemic thrombolysis (ST). (**A**) Pre-treatment CTPA demonstrating a large central pulmonary embolus with near-complete luminal obstruction. (**B**) Post-treatment CTPA following systemic thrombolysis showing partial reduction in thrombotic burden and improved, though incomplete, reperfusion of the pulmonary arteries. These images exemplify the radiologic effect of systemic thrombolysis in high-risk pulmonary embolism and illustrate the difference in anatomic clot clearance compared with surgical embolectomy.

The median duration of stay differed significantly between groups. Patients treated surgically experienced prolonged inpatient recovery with a mean hospital stay of 17.4 days (SD ±9.6), compared to 11.4 days (SD ±7.2) in the ST group (*p* < 0.001) (Table 2) (Figure 1 and Figure 2).

**Table 2:** Overview of Post Interventional Outcomes in ST and SPE Patients.

Outcomes	ST (*n* = 48)	SPE (*n* = 48)	*p*-Value
Overall Mortality	12	8	0.317
Pneumonia	9	12	0.461
Hemodynamic instability	5	12	0.063
Cardiogenic Shock	4	8	0.220
Continued right sided HF	1	5	0.093
Continued/new onset LV dysfunction	6	9	0.401
Post operative wound infection	0	1	0.999
AKI	4	8	0.219
Neurological deficit	6	1	0.05
Allergic reactions	1	0	0.999
Non-life threatening hemorrhage *	8	1	0.014
Life threatening hemorrhage	7	8	0.780
Hemothorax	0	1	0.999
Cardiac tamponade	0	7	0.006
Intracerebral hemorrhage	6	0	0.012
Multiorgan failure causing death	1	0	0.999
Post interventional ECMO **	6	7	0.767
Hospital length of stay (in days)	11.4 ± 7.2	17.4 ± 9.6	<0.001

* Neurological deficits include number of intracerebral hemorrhages. ** Number of post interventional ECMO excluded cases of pre-interventional ECMO.

## 4. Discussion

As individualized, risk-adapted strategies are increasingly emphasized in clinical practice, our retrospective institutional study provides insights into the effectiveness of surgical pulmonary embolectomy (SPE) compared to systemic thrombolysis (ST) in the management of high-risk patients with severe acute pulmonary embolism. Current evidence suggests that SPE is a viable alternative to ST and mortality rates are similar between treatments [4,8,9,20]. SPE may also offer improved pulmonary outcomes and a better complication profile, particularly in terms of neurological safety and bleeding risk [5,8,21].

This study provides further evidence supporting the role of SPE as a viable treatment option for high-risk acute pulmonary embolism, particularly in patients with significant hemodynamic compromise or contraindications to ST. Our findings align with recent literature indicating that SPE yields comparable mortality rates to ST, even in critically ill patients. A key observation in our analysis was the significantly lower incidence of neurological complications, particularly intracranial hemorrhage, among patients treated with SPE compared to those receiving ST. This finding is consistent with large-scale analyses, which have demonstrated higher rates of both major bleeding and intracranial hemorrhage in patients undergoing thrombolytic therapy [5,8,12,16,17,21]. While SPE carries inherent surgical risks, the risk of central nervous system hemorrhage is substantially lower, likely due to the avoidance of systemic fibrinolysis [8,17,21]. Consequently, the risk-benefit ratio of ST appears less favorable in patients with cerebrovascular vulnerability, such as older, frail patients with multiple comorbidities [22,23,24]. Our results therefore support the use of SPE as an effective alternative in patients at high bleeding risk or with absolute or relative contraindications to thrombolysis.

Although rates of severe hemorrhage were similar between groups, the nature and clinical implications of these events differed significantly. In the SPE cohort, bleeding complications were due to the surgical approach and generally manageable with standard operative or supportive measures. By contrast, ST was associated with diffuse and severe bleeding events, including intracranial hemorrhage, mucosal bleeding, and gastrointestinal hemorrhage, which are often challenging to control and frequently preclude further anticoagulation [5,8,10]. This highlights the procedural safety of SPE in selected patients and supports its role as a more controlled and predictable intervention in the management of high-risk PE.

The significantly longer hospital stay observed in the SPE group (mean 17.4 days vs. 11.4 days, *p* < 0.001) likely reflects the surgical nature of the procedure, the need for extended ICU monitoring, and the higher acuity of patients requiring ECMO support. These findings are in line with previous reports [5,15,20,25]. Importantly, this increased resource utilization must be considered alongside the potential for lower rates of disabling complications and reduced long-term morbidity. Notably, our surgical cohort included a higher proportion of patients requiring pre interventional va-ECMO support, which is independently associated with prolonged hospitalization and indicative of a more critically ill baseline population. The longer hospital stays seen with SPE reflects both the complexity of the procedure and the higher acuity of patients selected for surgery. While this increases resource use, it must be weighed against the potential for reduced long-term morbidity and better outcomes in high-risk, critically ill patients. The feasibility and safety of performing SPE in patients requiring preoperative ECMO deserve special mention. Our findings demonstrate that SPE can be successfully performed in this high-risk subgroup, aligning with growing evidence that ECMO-supported surgical embolectomy is a viable strategy in patients with refractory hemodynamic compromise [15]. The integration of ECMO with SPE offers hemodynamic stabilization and oxygenation, allowing definitive clot removal even in the most critically ill patients. The decision to proceed with SPE in such cases should be guided by institutional experience, surgical expertise, and the presence of a dedicated multidisciplinary team [18,26,27].

From a pathophysiological perspective, SPE offers several advantages over ST. Direct removal of embolic burden from the central pulmonary arteries results in immediate reduction in right ventricular afterload and restoration of forward flow [28]. This leads to more complete clearance of the pulmonary vasculature. In a comparative study of high-risk PE treatments, patients who underwent surgical embolectomy had significantly fewer residual perfusion defects on follow-up scans than those who received thrombolysis, 31% versus 76%, respectively [21,28]. This rapid reperfusion often translates into immediate hemodynamic improvement, a critical factor in unstable patients. Furthermore, surgical management allows for the removal of organized or pharmacologically resistant thrombi, which may not be effectively lysed by systemic therapy [28]. These findings suggest that surgery can eliminate thrombi that “fail to lyse” with drugs, including partly organized clots or those lodged in locations less accessible to catheter or systemic therapy. Moreover, surgical embolectomy allows extraction of hard, fibrous thrombus material (or concomitant right heart clots) that might not dissolve readily with pharmacologic fibrinolysis [21,28]. This advantage is especially pertinent when clots are subacute (beyond the ideal 48 h window for lysis) or when initial thrombolysis has failed. In such cases, proceeding to surgical embolectomy can achieve definitive clot removal and often rescue the patient.

Despite these benefits, SPE remains underutilized, likely due to limited surgical availability, concerns regarding operative risk, and lack of awareness of improved outcomes with contemporary techniques. The establishment of dedicated pulmonary embolism response teams (PERTs) may help address these barriers by fostering multidisciplinary collaboration and timely triage to surgical or catheter-based interventions. Our findings support the inclusion of SPE as a frontline consideration in the therapeutic algorithm for high-risk PE patients, particularly in centers with established cardiothoracic capabilities and ECMO support [4,18,29,30,31,32].

While dedicated Pulmonary Embolism Response Teams (PERTs) are essential to optimize patient triage and expand access to surgical and catheter-based therapies, recent advances in interventional radiological thrombectomy have further diversified the therapeutic landscape for acute high-risk PE. These techniques, including large-bore mechanical and aspiration systems, have recently emerged as non-lytic reperfusion modalities designed to restore pulmonary perfusion while minimizing bleeding risk.

Contemporary device trials and registry data have demonstrated rapid hemodynamic improvement, reflected by significant reductions in RV/LV ratio and low rates of major bleeding or intracranial hemorrhage. In the prospective FLARE trial using the FlowTriever system, mechanical thrombectomy achieved early hemodynamic recovery without adjunctive thrombolytics and with very low bleeding rates [33]. Comparable findings were observed in the EXTRACT-PE study employing the Indigo aspiration system, in which thrombolytics were avoided in more than 98% of patients and procedural safety was excellent [34]. Real-world evidence from the large FLASH registry confirmed these results, showing low 30-day mortality (<1%) and major adverse event rates around 2%, even among patients with lytic contraindications [35,36]. For hemodynamically unstable or high-risk PE, the FLAME study suggested that large-bore thrombectomy can be performed with acceptable mortality and safety profiles compared with historical data [37]. Most recently, the randomized PEERLESS trial compared mechanical thrombectomy to catheter-directed thrombolysis in intermediate–high-risk PE, demonstrating reduced clinical deterioration and post-procedure ICU utilization with similar mortality and bleeding [38].

Our results showed similar mortality but fewer neurological and non-life-threatening bleeding events with SPE compared to ST. Mechanical thrombectomy may therefore serve as a complementary, non-lytic reperfusion approach. It offers a balance between therapeutic efficacy and bleeding safety, particularly in centers where surgical options are limited. Unlike SPE, mechanical thrombectomy does not allow direct visual removal of organized or lysis-resistant central thrombus. Most available thrombectomy data are derived from intermediate-risk patient cohorts. Robust randomized evidence in high-risk populations is still limited. Consequently, patient selection between SPE and catheter thrombectomy should be individualized within a multidisciplinary PERT framework, considering clot morphology, bleeding risk, and institutional expertise [4,15,16,17,18,29,30,31,32,33,34,35,36,37,38].

### Limitations

This study has several limitations inherent to its retrospective, single-center design. Although baseline characteristics were well matched between groups, unmeasured confounding variables such as clot burden, RV dysfunction severity, and unidentified comorbidity indices may have influenced outcomes. Long-term follow-up data were not available and should be incorporated in future prospective analyses.

Additionally, no formal cost analysis was performed. While SPE involves greater upfront costs due to operative intervention and longer hospitalization, it is conceivable that the avoidance of hemorrhagic complications and recurrent embolic events could translate into cost savings over time.

## 5. Conclusions

Surgical pulmonary embolectomy is a safe and feasible alternative compared to systemic thrombolysis in the management of critically ill patients with severe acute pulmonary embolism. SPE comes with a lower incidence of neurologic and hemorrhagic complications, while having comparable rates of mortality. These findings underscore the importance of individualized, risk-adapted treatment pathways supporting the inclusion of SPE as a frontline consideration in the therapeutic algorithm for PE in critically ill patients, particularly in experienced centers with multidisciplinary support.

## Figures and Tables

**Table 1:** Patient Demographics and Risk Factors for pulmonary embolism (PE).

Patient Demographics	Overall (*n* = 96)	ST (*n* = 48)	SPE (*n* = 48)	*p*-Value
Baseline characteristics				
Age	65.8 ± 17	65.6 (24–101)	66.1 (31–93)	0.889
Male	48	27	21	0.223
Medical history				
Obesity	16	7	9	0.584
Atrial fibrillation (AF)	10	4	6	0.506
COPD (≥III)	3	1	2	0.557
Previous myocardial infarction	4	2	2	1.000
Previous malignancy	18	8	10	0.600
Smoking history	8	4	4	1.000
Chronic hypertension	35	18	17	0.832
Type 2 Diabetes mellitus	11	7	4	0.336
DVT	44	24	20	0.412
Pre-interventional CPR	27	16	11	0.256
Acute Cor Pulmonale with at least moderate * RV dysfunction	94	47	47	
Severe LV dysfunction	3	1	2	0.557
Pre-interventional va-ECMO	2	0	2	0.495
Localization of PE via CTPA				
Bilateral central	79	40	39	0.790
Bilateral segmental	1	1	0	0.999
Right sided central	3	2	1	0.5595
Left sided central	1	0	1	0.999
Left sided segmental	1	0	1	0.999
Peripheral	1	1	0	0.999
not specified	10	4	6	0.506

ST (Systemic thrombolysis); SPE (Surgical pulmonary embolectomy), DVT (deep vein thrombosis); va-ECMO (veno-arterial extracorporeal membrane oxygenation). * TAPSE 10–15 mm, S-prime (Sm) 6–9 cm/s.

## Data Availability

The original contributions presented in this study are included in the article. Further inquiries can be directed to the corresponding author.

## Abbreviations

The following abbreviations are used in this manuscript:

AKI	Acute Kidney Injury
CDT	Catheter Directed Thrombolysis
CTPA	Computed Tomography Pulmonary Angiography
DOAC	Direct Oral Anticoagulation
DVT	Deep Vein Thrombosis
ECMO	Extracorporeal Membrane Oxygenation
ICU	Intensive Care Unit
MACE	Major Adverse Cardiovascular Event
PA	Pulmonary Artery
PE	Pulmonary Embolism
PESI	Pulmonary Embolism Severity Index
PVR	Peripheral Vascular Resistance
RV	Right Ventricle
SPE	Surgical Pulmonary Embolectomy
sPESI	Simplified Pulmonary Embolism Severity Index
ST	Systemic Thrombolysis
VA-ECMO	Veno-Arterial ECMO
VV-ECMO	Veno-Venous ECMO

## References

[1] Barco S., Valerio L., Gallo A., Turatti G., Mahmoudpour S.H., Ageno W., Castellucci L.A., Cesarman-Maus G., Ddungu H., De Paula E.V. (2021). Global reporting of pulmonary embolism-related deaths in the World Health Organization mortality database: Vital registration data from 123 countries. Res. Pract. Thromb. Haemost..

[2] Bělohlávek J., Dytrych V., Linhart A. (2013). Pulmonary embolism, part I: Epidemiology, risk factors and risk stratification, pathophysiology, clinical presentation, diagnosis and nonthrombotic pulmonary embolism. Exp. Clin. Cardiol..

[3] Mojaddedi S., Jamil J., Bishev D., Essilfie-Quaye K., Elgendy I.Y. (2024). Risk Stratification and Management of Intermediate- and High-Risk Pulmonary Embolism. J. Clin. Med..

[4] Meneveau N. (2010). Therapy for acute high-risk pulmonary embolism: Thrombolytic therapy and embolectomy. Curr. Opin. Cardiol..

[5] Marti C., John G., Konstantinides S., Combescure C., Sanchez O., Lankeit M., Meyer G., Perrier A. (2015). Systemic thrombolytic therapy for acute pulmonary embolism: A systematic review and meta-analysis. Eur. Heart J..

[6] Choi J.H., O’mAlley T.J., Maynes E.J., Weber M.P., D’aNtonio N.D., Mellado M., West F.M., Galanis T., Gonsalves C.F., Marhefka G.D. (2020). Surgical pulmonary embolectomy outcomes for acute pulmonary embolism. Ann. Thorac. Surg..

[7] Yavuz S., Toktas F., Goncu T., Eris C., Gucu A., Ay D., Erdolu B., Tenekecioglu E., Karaagac K., Vural H. (2014). Surgical embolectomy for acute massive pulmonary embolism. Int. J. Clin. Exp. Med..

[8] QiMin W., LiangWan C., DaoZhong C., HanFan Q., ZhongYao H., XiaoFu D., XueShan H., Feng L., HuaBin C. (2020). Clinical outcomes of acute pulmonary embolectomy as the first-line treatment for massive and submassive pulmonary embolism: A single-centre study in China. J. Cardiothorac. Surg..

[9] Loyalka P., Ansari M.Z., Cheema F.H., Miller C.C., Rajagopal S., Rajagopal K. (2018). Surgical pulmonary embolectomy and catheter-based therapies for acute pulmonary embolism: A contemporary systematic review. J. Thorac. Cardiovasc. Surg..

[10] Pasrija C., Kronfli A., Rouse M., Raithel M., Bittle G.J., Pousatis S., Ghoreishi M., Gammie J.S., Griffith B.P., Sanchez P.G. (2018). Outcomes after surgical pulmonary embolectomy for acute submassive and massive pulmonary embolism: A single-center experience. J. Thorac. Cardiovasc. Surg..

[11] Kalra R., Bajaj N.S., Arora P., Arora G., Crosland W.A., McGiffin D.C., Ahmed M.I. (2017). Surgical Embolectomy for Acute Pulmonary Embolism: Systematic Review and Comprehensive Meta-Analyses. Ann. Thorac. Surg..

[12] Ishisaka Y., Watanabe A., Fujisaki T., Iwagami M., So M., Steiger D., Aoi S., Secemsky E.A., Wiley J., Kuno T. (2023). Comparison of interventions for intermediate to high-risk pulmonary embolism: A network meta-analysis. Catheter. Cardiovasc. Interv..

[13] Tan C.W., Balla S., Ghanta R.K., Sharma A.M., Chatterjee S. (2020). Contemporary management of acute pulmonary embolism. Semin. Thorac. Cardiovasc. Surg..

[14] Upadhyay H., Barnes J., Beattie A., Reicher J. (2024). Efficacy and safety of systemic thrombolysis and catheter-directed therapy in pulmonary embolism: A narrative review. Cureus.

[15] Treml B., Breitkopf R., Bukumirić Z., Bachler M., Boesch J., Rajsic S. (2022). ECMO predictors of mortality: A 10-year referral centre experience. J. Clin. Med..

[16] Konstantinides S.V., Meyer G., Becattini C., Bueno H., Geersing G.J., Harjola V.P., Huisman M.V., Humbert M., Jennings C.S., Jiménez D. (2020). 2019 ESC Guidelines for the diagnosis and management of acute pulmonary embolism developed in collaboration with the European Respiratory Society (ERS). Eur. Heart J..

[17] Goldberg J.B., Giri J., Kobayashi T., Ruel M., Mittnacht A.J.C., Rivera-Lebron B., DeAnda A., Moriarty J.M., MacGillivray T.E., on behalf of the American Heart Association (2023). Surgical Management and Mechanical Circulatory Support in High-Risk Pulmonary Embolism: A Scientific Statement From the American Heart Association. Circulation.

[18] Witkin A., Harshbarger S., Kabrhel C. (2016). Pulmonary embolism response teams. Semin. Thromb. Hemost..

[19] Bikdeli B., Khairani C.D., Bejjani A., Lo Y.-C., Mahajan S., Caraballo C., Jimenez J.V., Krishnathasan D., Zarghami M., Rashedi S. (2025). Validating International Classification of Diseases Code 10th Revision Algorithms for Accurate Identification of Pulmonary Embolism. J. Thromb. Haemost..

[20] Choi J.H., Lee S.Y., Park Y.H., Park J.H., Kim K.H. (2020). In-hospital outcome in patients who underwent extracorporeal membrane oxygenation in life-threatening high-risk pulmonary embolism. Int. J. Heart Fail..

[21] Lehnert P., Møller C.H., Mortensen J., Kjaergaard J., Olsen P.S., Carlsen J. (2017). Surgical embolectomy compared to thrombolysis in acute pulmonary embolism: Morbidity and mortality. Eur. J. Cardiothorac. Surg..

[22] Konstantinides S.V., Barco S., Lankeit M., Meyer G. (2016). Management of pulmonary embolism. J. Am. Coll. Cardiol..

[23] Martin C., Sobolewski K., Bridgeman P., Boutsikaris D. (2016). Systemic thrombolysis for pulmonary embolism: A review. Pharm. Ther..

[24] Califf R.M., Fortin D.F., Tenaglia A.N., Sane D.C. (1992). Clinical risks of thrombolytic therapy. Am. J. Cardiol..

[25] Cho Y.H., Sung K., Kim W.S., Jeong D.S., Lee Y.T., Park P.W., Kim D.K. (2016). Management of acute massive pulmonary embolism: Is surgical embolectomy inferior to thrombolysis?. Int. J. Cardiol..

[26] Davies M.G., Hart J.P. (2023). Current status of ECMO for massive pulmonary embolism. Front. Cardiovasc. Med..

[27] De Perrot M. (2022). Role of extracorporeal membrane oxygenation and surgical embolectomy in acute pulmonary embolism. Curr. Opin. Pulm. Med..

[28] Iaccarino A., Frati G., Schirone L., Saade W., Iovine E., D’aBramo M., De Bellis A., Sciarretta S., Greco E. (2018). Surgical embolectomy for acute massive pulmonary embolism: State of the art. J. Thorac. Dis..

[29] Serhal M., Haddadin I., Heresi G., Hornacek D., Shishehbor M., Bartholomew J. (2017). Pulmonary embolism response teams. J. Thromb. Thrombolysis..

[30] Dudzinski D., Piazza G. (2016). Multidisciplinary pulmonary embolism response teams. Circulation.

[31] Channick R.N. (2021). The Pulmonary Embolism Response Team: Why and How?. Semin. Respir. Crit. Care Med..

[32] Porres-Aguilar M., Rosovsky R.P., Rivera-Lebron B.N., Kaatz S., Mukherjee D., Anaya-Ayala J.E., Jimenez D., Jerjes-Sánchez C. (2022). Pulmonary embolism response teams: Changing the paradigm in the care for acute pulmonary embolism. J. Thromb. Haemost..

[33] Motiwala A., Tanwir H., Duarte A., Gilani S., DeAnda A., Zaidan M., Jneid H. (2024). Multidisciplinary approach to pulmonary embolism and the role of the pulmonary embolism response team. Curr. Cardiol. Rep..

[34] Tu T., Toma C., Tapson V.F., Adams C., Jaber W.A., Silver M., Khandhar S., Amin R., Weinberg M., Engelhardt T. (2019). A prospective, single-arm, multicenter trial of catheter mechanical thrombectomy for intermediate-risk pulmonary embolism (FLARE). JACC Cardiovasc. Interv..

[35] Sista A.K., Horowitz J.M., Tapson V.F., Rosenberg M., Elder M.D., Schiro B.J., Dohad S., Amoroso N.E., Dexter D.J., Loh C.T. (2021). Indigo aspiration system for treatment of acute pulmonary embolism (EXTRACT-PE). JACC Cardiovasc. Interv..

[36] Toma C., Jaber W.A., Weinberg M.D., Bunte M.C., Khandhar S., Stegman B., Gondi S., Chambers J., Amin R., Leung D.A. (2023). Acute outcomes for the full US cohort of the FLASH mechanical thrombectomy registry in pulmonary embolism. EuroIntervention.

[37] Khandhar S., Jaber W.A., Bunte M.C., Cho K.H., Weinberg M.D., Mina B., Stegman B., Pollak J., Khosla A., Elmasri F. (2023). Longer-term outcomes following mechanical thrombectomy for intermediate- and high-risk pulmonary embolism: 6-month FLASH registry results. J. Soc. Cardiovasc. Angiogr. Interv..

[38] Silver M.J., Gibson C.M., Giri J., Khandhar S., Jaber W., Toma C., Mina B., Bowers T., Greenspon L., Kado H. (2023). Outcomes in high-risk (massive) pulmonary embolism patients undergoing FlowTriever mechanical thrombectomy or other contemporary therapies: Results from the FLAME Study. Circ. Cardiovasc. Interv..

